# Clinical Outcomes, Incidence, Associated Factors, and Prediction Model of Intensive Care Unit Admission in Women With Postpartum Hemorrhage Following Cesarean Delivery: A Secondary Analysis of a Tertiary‐Care Cohort in Thailand

**DOI:** 10.1155/ogi/9946379

**Published:** 2026-08-10

**Authors:** Jitsupa Nithiuthai, Tripop Lertbunnaphong, Suneerat Kongsayreepong, Annop Piriyapatsom, Thanyarat Soponsiripakdee, Patchareya Nivatpumin

**Affiliations:** ^1^ Department of Anesthesiology, Faculty of Medicine Siriraj Hospital, Mahidol University, Bangkok, Thailand, mahidol.ac.th; ^2^ Department of Obstetrics and Gynecology, Faculty of Medicine Siriraj Hospital, Mahidol University, Bangkok, Thailand, mahidol.ac.th

**Keywords:** cesarean delivery, general anesthesia, intensive care unit admission, massive blood loss, perioperative outcomes, postpartum hemorrhage

## Abstract

**Objectives:**

We aimed to determine the clinical outcomes, incidence, associated factors, and a predictive model for intensive care unit (ICU) admission among patients with postpartum hemorrhage (PPH) following cesarean delivery.

**Study Design:**

Secondary analysis of a retrospective cohort of parturients with PPH following cesarean delivery between January 1, 2016, and December 31, 2020.

**Methods:**

Patients with an estimated blood loss < 1000 mL or incomplete data were excluded. Perioperative, obstetric, and anesthetic variables were evaluated. Multivariable logistic regression identified factors associated with ICU admission. Results are reported as adjusted odds ratios (aORs) with 95% confidence intervals (CIs).

**Results:**

Of 649 eligible patients, 44 (6.78%; 95% CI, 5.09–8.98) were admitted to the ICU. Estimated blood loss > 2200 mL (aOR 16.51; 95% CI, 7.28–37.45; *p* < 0.001) strongly predicted ICU admission. Other independent predictors were general anesthesia (aOR, 4.12; 95% CI, 1.76–9.65; *p* = 0.001) and gestational age < 36.2 weeks (aOR, 2.28; 95% CI, 1.11–4.67; *p* = 0.024). Emergency cesarean delivery was also predictive (aOR, 2.25; 95% CI, 1.02–4.92; *p* = 0.043). Among ICU admissions, 37/44 (84.1%) required mechanical ventilation. The median ICU length of stay was 1 day (IQR, 1‐2). ICU patients had higher rates of blood transfusion, hysterectomy, and reoperation than non‐ICU patients (all *p* < 0.001). An exploratory prediction model incorporating these variables demonstrated good discrimination (AUC 0.891; 95% CI 0.835–0.946).

**Conclusions:**

ICU admission was associated with more severe maternal illness and adverse perioperative outcomes. Factors associated with ICU admission included severe blood loss, general anesthesia, earlier gestational age, and emergency cesarean delivery. Recognition of these characteristics may facilitate timely escalation of monitoring and multidisciplinary management when severe PPH occurs.

**Trial Registration:** ClinicalTrials.gov identifier: NCT04833556

**Highlights**
• Approximately 7% of patients with postpartum hemorrhage after cesarean delivery required intensive care unit (ICU) admission.
• Independent predictors of ICU admission were estimated blood loss > 2200 mL, general anesthesia, gestational age < 36.2 weeks, and emergency cesarean delivery.
• ICU patients had markedly higher rates of blood transfusion, hysterectomy, and reoperation than non‐ICU patients.
• Early identification of high‐risk parturients is essential for adequate perioperative preparation, timely intervention, and improved clinical outcomes.

## 1. Introduction

Postpartum hemorrhage (PPH) remains a leading global contributor to maternal morbidity and mortality, with a reported prevalence of approximately 0.4%–5.1% [1, 2]. Siriraj Hospital, a tertiary referral center in Bangkok, Thailand, has an annual delivery volume of 7500–8000 births. There, the prevalence of PPH after cesarean delivery between 2016 and 2020 was 3.8% [3]. Massive hemorrhage and the subsequent need for massive blood transfusion can cause multiorgan complications. These include disseminated intravascular coagulation, congestive heart failure, acute kidney injury, intensive care unit (ICU) admission, prolonged hospital stays, and increased healthcare costs [4–7].

A previous study of patients with massive PPH reported that 15 of 21 (71.4%) required ICU admission. Among these ICU patients, 12 developed disseminated intravascular coagulation and 2 (9%) died [6]. Regarding long‐term outcomes, maternal ICU admission was associated with increased 1‐year hospital readmission and mortality, as well as higher odds of stillbirth and neonatal critical‐care admission [8]. The pattern of ICU admission varies widely across countries, reflecting differences in obstetric care standards, resource availability, and clinical management protocols [6, 9, 10]. Numerous studies have identified predictors of maternal outcomes. These include scoring systems, such as the Acute Physiology and Chronic Health Evaluation II (APACHE II) and Sequential Organ Failure Assessment (SOFA) scores. Other predictors include ICU length of stay and shock index among general ICU patients [9–11]. However, research focusing specifically on risk factors or predictors of ICU admission in obstetric patients remains limited.

Therefore, this study aimed to determine the incidence, clinical outcomes, and factors associated with ICU admission among patients with PPH following cesarean delivery at the largest university hospital in Thailand. An improved understanding of ICU utilization and the characteristics associated with critical care requirements may support timely escalation of monitoring and multidisciplinary management when severe PPH occurs.

## 2. Methods

### 2.1. Study Design and Setting

This single‐center retrospective cohort study was conducted at Siriraj Hospital, Bangkok, Thailand. We performed a secondary analysis of a previously published cohort of patients who underwent cesarean delivery complicated by PPH between January 1, 2016, and December 31, 2020 [3]. In the original study, PPH was identified using the International Classification of Diseases, Tenth Revision code O72.1. That study focused on the epidemiology, etiologies, perioperative characteristics, and hemorrhage‐related outcomes of PPH.[3] The present study addresses a distinct question, evaluating ICU utilization, ICU‐related clinical outcomes, and factors associated with ICU admission among patients with PPH following cesarean delivery.

All study procedures complied with International Council for Harmonization—Good Clinical Practice Guidelines and the ethical principles of the Declaration of Helsinki and the Belmont report. The study protocol was approved by the Siriraj Institutional Review Board (protocol number 005/2564, approval number Si‐161/2021). This article follows the Strengthening the Reporting of Observational Studies in Epidemiology reporting guidelines.

### 2.2. Study Population

The inclusion criterion was cesarean delivery with a diagnosis of PPH during the study period. The exclusion criteria were a gestational age of less than 24 weeks, blood loss of less than 1000 mL within the first 24 h after delivery, or incomplete medical records.

### 2.3. Data Collection

Clinical data, including preoperative conditions, intraoperative management, postoperative outcomes, and ICU‐related information, were extracted from electronic medical records. For patients admitted to the ICU, details on the indications for admission, interventions, monitoring, and treatment were also collected. The APACHE II and SOFA scores were recorded.

### 2.4. Definitions

PPH was defined as blood loss ≥ 1000 mL within 24 h of cesarean delivery.[7] Etiologies of PPH were categorized as follows: uterine atony (requiring ≥ 2 uterotonic agents); abnormal placentation (placenta previa or placenta accreta spectrum); trauma (injury to the uterus or surrounding internal organs); and coagulopathy (documented abnormal coagulation parameters).

Estimated blood loss was routinely assessed by the clinical care team using several methods. These were suction canister measurements (after accounting for irrigation fluid), calibrated collection drapes with volume markings, and visual assessment of blood absorbed by surgical gauzes and drapes. The estimated blood loss documented in the anesthesia and operative records was used for analysis.

ICU admission was defined as admission to the surgical or medical ICU after delivery. At our institution, ICU admission is determined jointly by the attending ICU physician and the perioperative care team, based primarily on clinical judgment. The decision takes into account the patient’s hemodynamic status, overall clinical condition, anticipated postoperative monitoring requirements, and ICU bed availability. Laboratory and physiologic data, including blood gas analysis and other investigations, may contribute to decision‐making but are not used as standalone admission criteria.

During the study period, no standardized institutional criteria for ICU admission following PPH were in place. Because ICU beds were limited, admission decisions followed a prioritization model that gave priority to patients with the greatest anticipated need for intensive monitoring or organ support. Common indications for ICU admission included hemodynamic instability requiring vasopressor support, massive transfusion, need for mechanical ventilation, persistent cardiorespiratory instability, or anticipated high‐risk postoperative monitoring.

Hypotension was defined as an intraoperative or postoperative systolic blood pressure < 90 mmHg or a reduction ≥ 20% from the preanesthetic baseline. Transfusion‐related acute lung injury was defined as new‐onset acute lung injury within 6 h of transfusion, with other causes ruled out [12]. Acute kidney injury was defined according to the Kidney Disease: Improving Global Outcomes 2012 guidelines and was based on elevated serum creatinine or reduced urine output [13]. Neonatal birth asphyxia was defined as an Apgar score of less than 7 at 5 min.

### 2.5. Statistical Analysis

All analyses were performed using IBM SPSS Statistics version 29 (IBM Corp, Armonk, NY, USA). Data are presented as counts (percentages), means (SD), medians (IQR), or minimum–maximum values, as appropriate. Categorical variables were compared using the chi‐square test or Fisher exact test. Continuous variables were analyzed using the independent‐samples *t* test or the Mann–Whitney *U* test. Receiver operating characteristic (ROC) curves were used to determine cutoff values for gestational age and estimated blood loss that best predicted ICU admission. Factors associated with ICU admission were first identified by univariable analysis, followed by forward stepwise binary logistic regression for the multivariable analysis. All variables hypothesized to be associated with critically adverse outcomes were included in the multivariable analysis. To address potential multicollinearity, variance inflation factors were calculated for all independent variables, and any variable with a value > 10 was excluded.

The model’s discrimination for predicting ICU admission was evaluated using the area under the ROC curve (AUC). Calibration was assessed using the Hosmer–Lemeshow goodness‐of‐fit test, in which a nonsignificant result (*p* > 0.05) indicated adequate calibration. The relationship between the SOFA score and ICU length of stay was assessed using the Spearman correlation coefficient (rho). A 95% confidence interval for the incidence of ICU admission was calculated using Wilson’s method. The 95% confidence intervals for positive and negative predictive values were calculated using the standard logit method. A two‐sided *p* value < 0.05 was considered statistically significant.

## 3. Results

### 3.1. Incidence and Participant Flow

**FIGURE 1:**
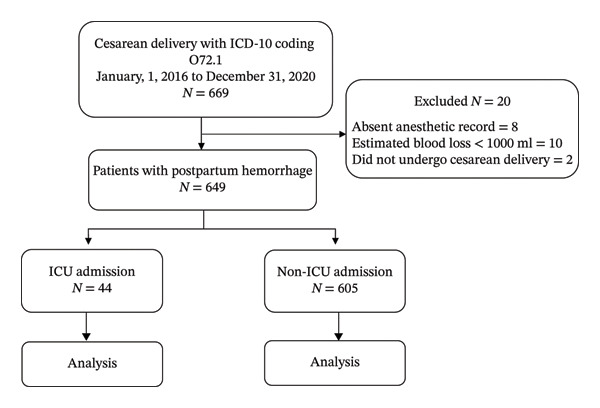
Study flow diagram showing cohort selection of parturients with postpartum hemorrhage (ICD‐10 O72.1) after cesarean delivery and their allocation to ICU versus non‐ICU groups from January 1, 2016, to December 31, 2020. Abbreviations: ICD‐10, International Classification of Diseases, Tenth Revision; ICU, intensive care unit.

During the study period, there were 17,187 cesarean deliveries. Among these, 649 patients met the study criteria for PPH and 44 (6.78%) were admitted to the ICU (95% CI 5.09–8.98). Almost all were admitted to the surgical ICU; only 1 patient required the cardiac ICU, owing to intraoperative supraventricular tachycardia. The study flow is presented in Figure 1.

### 3.2. Baseline and Operative Characteristics

Demographic characteristics and preoperative data for PPH patients admitted and not admitted to the ICU are compared in Table 1. The ICU and non‐ICU groups did not differ in age, parity, multiple pregnancy, pregnancy‐induced hypertension, gestational diabetes, or a history of antepartum hemorrhage. The mean gestational age was lower in the ICU group than in the non‐ICU group (*p* = 0.001). A preoperative diagnosis of abnormal placentation was made in 22 patients (50%) in the ICU group compared with 173 (28.6%) in the non‐ICU group (*p* = 0.003).

**TABLE 1:** Baseline demographic, obstetric, and preoperative characteristics of women undergoing cesarean delivery complicated by postpartum hemorrhage, stratified by ICU admission (*N* = 649).

Characteristic	ICU admission *n* = 44	Non‐ICU admission *n* = 605	*p* value
Age (years)	34.3 ± 5.7 (23–43)	33.8 ± 5.6 (17–48)	0.586
BMI (kg/m^2^)	26.8 ± 4.7 (19.5–38.7)	29.4 ± 4.8 (19.1–48.9)	0.001^∗^
Primigravida	16 (36.4)	275 (45.5)	0.242
Gestational age (weeks)	35.1 ± 4.1 (24.1–41.3)	37.3 ± 2.6 (25.2–42.1)	0.001^∗^
ASA classification			
II	33 (75.0)	542 (89.6)	0.006^∗^
III	11 (25.0)	61 (10.1)	
IV	0 (0)	2 (0.3)	
Emergency cesarean delivery	29 (65.9)	380 (62.8)	0.681
Multiple pregnancy			
Twin	9 (20.5)	63 (10.4)	0.070
Triplet	0 (0)	2 (0.3)	
Pregnancy‐induced hypertension	3 (6.8)	94 (15.5)	0.117
Gestational diabetes	6 (13.6)	105 (17.4)	0.527
History of antepartum hemorrhage	11 (25.0)	98 (16.2)	0.132
Preoperative diagnosis of abnormal placentation	22 (50.0)	173 (28.6)	0.003^∗^

*Note:* Values are mean ± SD (range) or number (%).

Abbreviations: ASA, American Society of Anesthesiologists physical‐status classification; BMI, body mass index; ICU, intensive care unit; SD, standard deviation.

^∗^ *p* < 0.05 indicates statistical significance.

Intraoperative and postoperative data are shown in Table 2. In ICU‐admitted patients, abnormal placentation was the predominant etiology of PPH (45.5%), followed by uterine atony (43.2%) and internal organ injury (11.4%). In contrast, uterine atony was the primary cause of PPH in non‐ICU patients (64.1%), followed by abnormal placentation (28.1%). ICU‐admitted patients also had greater median blood loss than non‐ICU patients (*p* < 0.001). In addition, they received larger volumes of fluids and blood products during both the intraoperative and postoperative periods (all *p* < 0.001). Rates of intraoperative interventions, including hysterectomy, uterine artery ligation, and repeat operations within 24 h, were also higher among ICU patients. Critically ill obstetric patients had a longer median hospital stay than non‐ICU patients (9 [IQR 6.3–14.8] days vs 5 [IQR 4–7] days; *p* < 0.001).

**TABLE 2:** Intraoperative management and early postoperative outcomes in cesarean delivery complicated by postpartum hemorrhage, stratified by ICU admission (*N* = 649).

Characteristic	ICU admission *n* = 44	Non‐ICU admission *n* = 605	*p* value
Intraoperative data			
Choice of anesthesia^1^			
General anesthesia	35 (79.5)	180 (29.8)	< 0.001^∗^
Regional anesthesia	9 (20.5)	425 (70.2)	
Primary cause of PPH			
Uterine atony	19 (43.2)	388 (64.1)	0.032^∗^
Abnormal placentation	20 (45.5)	170 (28.1)	
Injury to internal organ(s)	5 (11.4)	39 (6.4)	
Coagulopathy	0 (0)	8 (1.3)	
Intraoperative blood loss (mL)	3600 [2500–5500]	1300 [1000–1700]	< 0.001^∗^
Total volume of crystalloid fluid (mL)	3250 [1925–4500]	1800 [1300–2420]	< 0.001^∗^
Intraoperative blood transfusion	38 (86.4)	226 (37.4)	< 0.001^∗^
Intraoperative hypotension	38 (86.4)	463 (76.5)	0.133
Hysterectomy	32 (72.7)	74 (12.2)	< 0.001^∗^
Uterine artery ligation	8 (18.2)	25 (4.1)	< 0.001^∗^
Intrauterine balloon insertion	7 (15.9)	34 (5.6)	0.016
B‐Lynch suture	2 (4.5)	9 (1.5)	0.167
Neonatal birth asphyxia	8 (18.2)	27 (4.5)	< 0.001^∗^
Postoperative data			
Reoperation within 24 h	16 (36.4)	16 (2.6)	< 0.001^∗^
Maternal hypotension	27 (61.4)	66 (10.9)	< 0.001^∗^
Maternal hemorrhage	21 (47.7)	77 (12.7)	< 0.001^∗^
Maternal anemia	32 (72.7)	329 (54.4)	0.018^∗^
Postoperative blood transfusion	27 (61.4)	134 (22.1)	< 0.001^∗^
Maternal death	1 (2.3)	0 (0)	0.068
Hospital length of stay (days)	9 [6.3–14.8]	5 [4–7]	< 0.001^∗^

*Note:* Values are median [IQR], mean ± SD, or number (%), as appropriate.

Abbreviations: ICU, intensive care unit; IQR, interquartile range; PPH, postpartum hemorrhage; SD, standard deviation.

^1^“General anesthesia” comprises sole general anesthesia, general anesthesia + epidural analgesia, or conversion from regional to general anesthesia; “Regional anesthesia” comprises single‐shot spinal, sole epidural, or combined spinal‐epidural techniques.

^∗^ *p* < 0.05 indicates statistical significance.

### 3.3. Intensive Care Course and Outcomes

Patient characteristics and outcomes in the ICU are presented in Table 3. Most patients required initial respiratory support, predominantly mechanical ventilation (84.1%), and hemodynamic support with continuous intravenous vasopressors (34.1%). However, these interventions were successfully weaned and discontinued within the first 24 h in most patients. Three patients (6.8%) were intubated for more than 72 h, with 1 remaining intubated for 5 days. Two patients had a ruptured uterus and required massive blood transfusions. Among ICU‐admitted patients, there were no cases of perioperative myocardial infarction, stroke, or thromboembolism. Three patients experienced cardiac arrest, one of whom did not survive.

**TABLE 3:** Clinical course and outcomes of patients requiring ICU admission after cesarean delivery complicated by postpartum hemorrhage (*N* = 44).

Clinical characteristics and outcomes	Value
SOFA score at day 0	5.0 ± 2.9
SOFA score at day 1	1.7 ± 1.5
APACHE II score at day 0	11.4 ± 4.7 (3–30)
Blood transfusion	27 (61.4)
FFP transfusion	16 (36.4)
Platelet transfusion	8 (18.2)
Disseminated intravascular coagulation	2 (4.5)
Vasopressor or inotropic infusion	15 (34.1)
Ventilator support	37 (84.1)
Prolonged intubation (> 72 h)	3 (6.8)
High‐flow oxygen therapy	1 (2.3)
Postoperative pulmonary complications (pulmonary edema)	1 (2.3)
Serious arrhythmia (supraventricular tachycardia)	1 (2.3)
Transfusion‐related acute lung injury	5 (11.4)
Acute kidney injury	1 (2.3)
Cardiac arrest	3 (6.8)
Length of ICU stay (days)	1 [1–2]
Length of hospital stay (days)	9 [6.3–14.8]
Maternal death	1 (2.3)

*Note:* Values are mean ± SD (range), median [IQR], or number (%), as appropriate.

Abbreviations: APACHE II, Acute Physiology and Chronic Health Evaluation II; FFP, fresh frozen plasma; ICU, intensive care unit; SD, standard deviation; SOFA, Sequential Organ Failure Assessment.

The SOFA score on the first day of ICU admission correlated positively with ICU length of stay (Spearman’s rho = 0.379; *p* = 0.011). The mean APACHE II score was 11.4 ± 4.7 (range, 3–30). The median ICU length of stay was 1 day (IQR, 1–2), and the median hospital length of stay was 9 days (IQR, 6.3–14.8).

### 3.4. Factors Associated With ICU Admission

Univariable and multivariable logistic regression analyses identified several factors associated with ICU admission after delivery, as shown in Table 4. ROC curve analysis of gestational age and estimated blood loss was used to determine optimal cutoff values for predicting ICU admission. In the multivariable analysis, estimated blood loss > 2200 mL, general anesthesia, gestational age < 36.2 weeks, and emergency cesarean delivery were independently associated with ICU admission.

**TABLE 4:** Logistic‐regression analysis of factors independently associated with ICU admission following cesarean delivery complicated by postpartum hemorrhage.

Variable	ICU admission *N* = 44	Non‐ICU admission *N* = 605	Crude OR (95% CI)	*p* value	Adjusted OR (95% CI)	*p* value	VIF	*β* coefficient
Age ≥ 35 years	22 (50.0)	301 (49.8)	1.01 (0.55–1.86)	0.975				
Gestational age < 36.2 weeks^1^	25 (56.8)	141 (23.3)	4.33 (2.32–8.10)	< 0.001^∗^	2.28 (1.11–4.67)	0.024^∗^	1.110	0.824
Emergency cesarean delivery	29 (65.9)	380 (62.8)	1.15 (0.60–2.18)	0.681	2.25 (1.02–4.92)	0.043^∗^	1.049	0.809
ASA classification III and IV	11 (25.0)	63 (10.4)	2.87 (1.38–5.95)	0.005^∗^				
History of antepartum hemorrhage	11 (25.0)	98 (16.2)	1.72 (0.84–3.52)	0.136				
Blood loss > 2200 mL^2^	35 (79.5%)	84 (13.9%)	24.12 (11.19–51.99)	< 0.001^∗^	16.51 (7.28–37.45)	< 0.001^∗^	1.155	2.804
General anesthesia^3^	35 (79.5)	180 (29.8)	9.18 (4.32–19.49)	< 0.001^∗^	4.12 (1.76–9.65)	0.001^∗^	1.186	1.416
Primary cause of PPH: abnormal placentation	20 (45.5)	170 (28.1)	2.12 (1.03–4.36)	0.040^∗^				

*Note:* Values are number (%).

Abbreviations: ASA, American Society of Anesthesiologists physical‐status classification; confidence interval; ICU, intensive care unit; OR, odds ratio; PPH, postpartum hemorrhage; VIF, variance inflation factor.

^1^Gestational‐age threshold (< 36.2 weeks) derived from receiver operating characteristic curve analysis, with area under the curve = 0.689 (*p* < 0.001).

^2^Blood‐loss threshold (> 2200 mL) derived from receiver operating characteristic curve analysis, with area under the curve = 0.839 (*p* < 0.001).

^3^“General anesthesia” comprises sole general anesthesia, general anesthesia + epidural analgesia, or conversion from regional to general anesthesia.

^∗^ *p* < 0.05 indicates statistical significance.

These four variables were subsequently incorporated into an exploratory prediction model for ICU admission. For each patient, presence of a factor was scored as 1 and absence as 0. The prediction score was weighted by the regression coefficients (*β* coefficients) from the multivariable logistic regression. The resulting scoring system was

Prediction score = [gestational age < 36.2 weeks × 1] + [emergency condition × 1] + [estimated blood loss > 2200 mL × 3] + [general anesthesia × 1.5].

### 3.5. Prediction Model Performance

**FIGURE 2:**
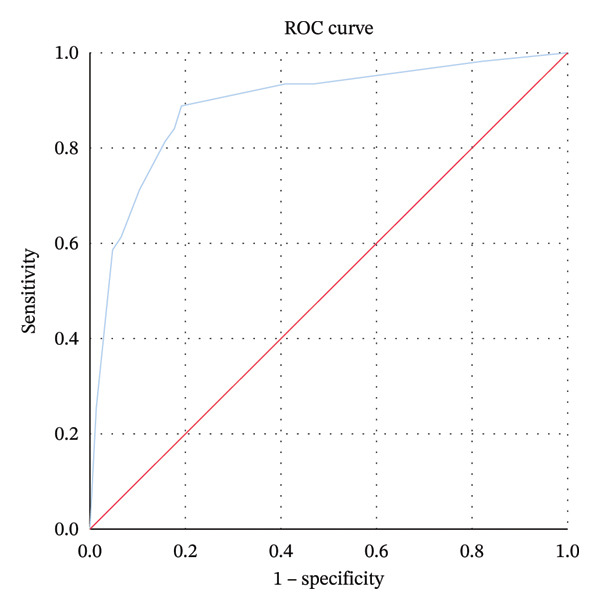
Receiver operating characteristic curve for the prediction model (cutoff score 2.5). The prediction score was calculated as gestational age (< 36.2 weeks) × 1 + emergency condition × 1 + estimated blood loss (> 2200 mL) × 3 + general anesthesia × 1.5. The area under the curve (AUC) was 0.891 (95% confidence interval 0.835–0.946; *p* < 0.001). Abbreviations: AUC, area under the curve; ROC, receiver operating characteristic.

The predictive performance of the model is presented in Table 5. The model showed excellent discrimination, with an AUC of 0.891 (95% CI 0.835–0.946). The Hosmer–Lemeshow goodness‐of‐fit test did not indicate evidence of poor model fit (*p* = 0.632). The ROC curve is shown in Figure 2. At the selected cutoff of > 2.5, the model had a sensitivity of 88.6%, a specificity of 81.2%, a positive predictive value of 25.5% (95% CI, 21.9%–29.4%), and a negative predictive value of 99.0% (95% CI, 97.7%–99.6%).

**TABLE 5:** Predictive performance of the prediction model at a cutoff score of 2.5.

Predictive performance	Value (95% confidence interval)
AUC	0.891 (0.835–0.946)
Accuracy	81.7% (78.5%–84.6%)
Sensitivity	88.6% (75.4%–96.2%)
Specificity	81.2% (77.8%–84.2%)
Positive predictive value	25.5% (21.9%–29.4%)
Negative predictive value	99.0% (97.7%–99.6%)

*Note:* AUC, area under the receiver operating characteristic curve.

## 4. Discussion

This retrospective cohort study evaluated the incidence, clinical outcomes, and factors associated with ICU admission among parturients with PPH following cesarean delivery. The study was conducted at a tertiary university hospital in Thailand over a 5‐year period ending in 2020. Of 649 women with PPH following cesarean delivery, 44 (6.78%) required ICU admission. On multivariable analysis, the factors independently associated with ICU admission were estimated blood loss > 2200 mL, general anesthesia, gestational age < 36.2 weeks, and emergency cesarean delivery.

### 4.1. Rate of ICU Admission

The rate of ICU admission among cesarean delivery patients with PPH at our institution differed from rates reported worldwide. For example, the reported rate in India was 71.4% [6]. The large discrepancy in ICU admission rates can be attributed to several factors. First, differences in obstetric care standards, such as protocols for monitoring, diagnosing, and managing pregnancy‐related conditions, affect thresholds for ICU admission. Second, resource availability, including specialized personnel, advanced equipment, and ICU bed capacity, may substantially affect clinical decision‐making. Facilities with limited resources often reserve ICU care for only the most critically ill patients. Third, variations in clinical management practices, including the timing of interventions, stabilization strategies, and multidisciplinary coordination, may also contribute to differences in ICU utilization [10].

Most ICU patients required ICU‐level organ‐supportive therapies, including mechanical ventilation (84.1%) and vasopressor support (34.1%), indicating a genuine need for critical care despite a median ICU length of stay of 1 day (IQR, 1–2). The relatively short duration of ICU admission likely reflects successful early stabilization following definitive hemorrhage control, blood product resuscitation, correction of coagulopathy, and short‐term organ support.

Our ICU admission rate was notably higher than the pooled rate of 2.8% reported in low‐ and middle‐income countries in a large meta‐analysis by Tripathy et al. [14] In addition to differences in obstetric care standards and resource availability, this discrepancy may reflect institutional differences in postoperative monitoring and ICU utilization. At our center, such monitoring is provided for patients with severe PPH who require close observation, ventilatory support, or ongoing hemodynamic management.

### 4.2. Factors Associated With ICU Admission

#### 4.2.1. Massive Blood Loss

Median intraoperative blood loss was markedly higher among patients admitted to the ICU than among those managed without ICU admission. In the multivariable analysis, estimated blood loss exceeding 2200 mL was the factor most strongly associated with postoperative ICU admission. Nevertheless, this association should be interpreted cautiously. Excessive blood loss likely reflects greater perioperative disease severity and physiological instability, which necessitate intensive postoperative monitoring and support, rather than being an independent causal determinant of ICU admission. In addition, the magnitude of the aOR should be interpreted cautiously, given the wide confidence interval and the limited number of ICU admissions. Therefore, substantial hemorrhage may be better regarded as a surrogate indicator of clinical complexity and hemodynamic compromise that influences the decision for ICU admission.

Additionally, ICU patients developed marked postoperative hypotension and anemia, required larger volumes of crystalloids and blood products, and needed vasopressor or inotropic support. These patients also required respiratory support with either mechanical ventilation or high‐flow oxygen therapy. They developed more transfusion‐related complications, such as transfusion‐related acute lung injury and pulmonary edema.

The prevalence of transfusion‐related acute lung injury among our ICU patients was 11.4%. This is comparable to the 11.03% reported in a Mexican study by Martínez‐Martínez et al., who identified massive transfusion as a major risk factor for such injury [15]. A study from China also reported that intraoperative blood transfusion during cesarean delivery was associated with elevated systemic inflammatory markers, increased postoperative complication rates, and prolonged hospital stays [16]. Consequently, strategies to minimize blood loss and limit transfusion should be implemented to reduce the risk of maternal mortality, massive transfusion, and associated complications. These strategies include early detection through quantitative blood loss measurement and early administration of tranexamic acid (within 3 h of PPH, as recommended by the International Federation of Gynecology and Obstetrics in 2022) [4].

#### 4.2.2. Gestational Age

Abnormal placentation often necessitates cesarean delivery at an earlier gestational age and the use of general anesthesia because of anticipated surgical complexity [17]. In our study, 50% of patients admitted postoperatively to the ICU had a preoperative diagnosis of abnormal placentation and presented at lower gestational ages. In our analysis, a gestational age threshold of < 36.2 weeks was independently associated with ICU admission. Lower gestational age may instead reflect underlying high‐risk obstetric conditions, such as placenta accreta spectrum, placental abruption, or severe hypertensive disorders of pregnancy. These conditions often necessitate earlier delivery and are themselves associated with increased maternal morbidity.

Although abnormal placentation was associated with ICU admission in the univariable analysis, this association was no longer statistically significant after adjustment for hemorrhage severity and perioperative factors in the multivariable model. This finding suggests that the increased likelihood of ICU admission with abnormal placentation may be mediated through greater blood loss, increased surgical complexity, and the need for perioperative interventions. The diagnosis itself may not be the direct cause.

Our findings are consistent with the American College of Obstetricians and Gynecologists recommendation that cesarean delivery for placenta accreta spectrum be scheduled between 34 weeks and 35 weeks 6 days of gestation. This timing balances the risks of prematurity against surgical preparedness [17, 18]. From a physiological standpoint, preterm delivery has been hypothesized to increase uterine bleeding because of an underdeveloped lower uterine segment and reduced oxytocin receptor sensitivity [19]. Lanza et al. reported that, in a Brazilian obstetric ICU, gestational age < 34 weeks was associated with severe maternal outcomes [10]. This relationship was attributed to the higher incidence of preeclampsia with severe features and placental abruption, conditions that increase PPH risk and often necessitate early delivery. Consistent with these findings, Blanc et al. reported a strong association between delivery at < 26 weeks and severe maternal morbidity [20, 21]. In contrast, Butwick et al. found that women delivering at 41‐42 weeks of gestation faced the highest PPH risk [20, 21]. Despite conflicting evidence on gestational age as a maternal risk factor, the most recent systematic review and meta‐analysis of PPH causes and risk factors did not include preterm delivery or gestational age [22].

#### 4.2.3. Choice of Anesthesia

Anesthetic technique is influenced by surgical type and complexity, patient comorbidities or emergencies, and fetal considerations. General anesthesia (alone or combined with neuraxial techniques) is typically reserved for patients with anticipated perioperative complications or maternal instability. Borovac‐Pinheiro et al. reported that spinal or epidural anesthesia did not affect postpartum bleeding after vaginal delivery [23]. Chang et al. investigated whether anesthetic management is a risk factor for PPH following cesarean delivery. They adjusted for numerous maternal factors, including maternal age, placental complications (abruption, previa, accreta), an overdistended uterus, pregnancy‐induced hypertension, prolonged labor, uterine myoma, prior myomectomy, and previous or emergent cesarean delivery, as well as fetal factors such as parity and gestational age. After these adjustments, general anesthesia was associated with an approximately 8.15‐fold increase in the odds of cesarean PPH compared with spinal or epidural anesthesia [24].

In our study, almost 80% of ICU‐admitted patients received general anesthesia, reflecting its anticipated use in more complex cases with heavy bleeding, such as abnormal placentation. In the multivariable analysis, general anesthesia was independently associated with ICU admission. Munoz et al. found that converting from neuraxial to general anesthesia yields intraoperative outcomes comparable to, and postoperative outcomes better than, primary general anesthesia in cesarean hysterectomy for placenta accreta spectrum. While ICU admission rates and ICU length of stay were similar between groups, the total postoperative length of stay was shorter with neuraxial anesthesia (3.76 vs 6.35 days) [25].

Despite theoretical concerns that inhalational maintenance anesthesia may impair uterine contractility, PPH in our ICU cohort was nearly equally attributable to abnormal placentation and uterine atony. In contrast, uterine atony remained the predominant cause of PPH in non‐ICU patients (64.1%), even though nearly three‐quarters of them received regional anesthesia.

#### 4.2.4. Emergency Surgery

Emergency care in patients with a high comorbidity burden often leads to unplanned ICU admission. Such admissions are associated with increased mortality, prolonged hospital stays, and higher costs [26]. Lucidi et al. reported that emergency cesarean delivery occurs in approximately 35% of pregnancies complicated by placenta accreta spectrum and is associated with adverse maternal and neonatal outcomes [27]. Compared with scheduled cesarean delivery, emergency cesarean delivery in placenta accreta spectrum is associated with greater intraoperative blood loss and a greater need for packed red blood cells and blood products. ICU admission rates are also higher [27]. Our results align with an observational study by Munoz et al. on predictors of ICU admission after cesarean hysterectomy for placenta accreta spectrum [28]. In that study, patients requiring ICU admission were more likely to deliver at an earlier gestational age, experience more than two episodes of vaginal bleeding, and undergo emergency delivery.

#### 4.2.5. Clinical Implications

The factors associated with ICU admission in this study may have practical implications for the perioperative management of patients with PPH following cesarean delivery. Although some factors, particularly estimated blood loss, likely reflect the severity of ongoing hemorrhage rather than preoperative predictors, recognition of these characteristics may facilitate earlier escalation of care when severe PPH develops. In high‐risk situations, such as abnormal placentation, preterm delivery, emergency cesarean delivery, or substantial blood loss, multidisciplinary coordination among obstetricians, anesthesiologists, intensivists, and transfusion services may be warranted. Potential interventions include early blood bank notification, preparedness for massive transfusion, consideration of invasive hemodynamic monitoring, and planning for postoperative ICU or high‐dependency unit observation when clinically indicated.

The exploratory prediction model showed high sensitivity and negative predictive value. This suggests good performance in identifying patients unlikely to require ICU admission within this cohort. However, the relatively low positive predictive value indicates that a substantial proportion of patients classified as high risk did not ultimately require ICU admission. Accordingly, the model should be considered hypothesis‐generating and may help support risk stratification and clinical decision‐making in conjunction with clinical judgment. External validation is warranted before its use for ICU triage or resource allocation in routine practice.

### 4.3. Clinical Outcomes

#### 4.3.1. Maternal Outcomes

The prolonged hospital stays in our study reflect greater illness severity among ICU‐admitted patients. These patients experienced increased blood loss, received larger volumes of resuscitative fluids and blood products, and were more likely to require hysterectomy, uterine artery ligation, or reoperation within 24 h of cesarean delivery. Most ICU patients required organ‐supportive therapies, including mechanical ventilation (84.1%) and vasopressor support (34.1%). Most ICU patients required respiratory or hemodynamic support for less than 24 h. However, the median ICU length of stay was 1 day, indicating that most patients required only a brief period of intensive care. This likely reflects successful early stabilization following hemorrhage control, blood product resuscitation, correction of coagulopathy, and short‐term organ support.

On ICU admission, the SOFA score (an established tool for quantifying organ dysfunction and illness severity) correlated positively with ICU length of stay. This finding underscores its value for early severity assessment in obstetric critical care. Owing to the low overall mortality (1 death among 44 ICU patients), the statistical power to evaluate the APACHE II score for mortality prediction was limited. A 22‐year review by Yi et al. at a tertiary care center evaluated the APACHE II score for predicting maternal mortality. The score performed better among patients admitted to the ICU for PPH than in the general obstetric ICU population [29]. These findings suggest that, although the APACHE II score has limited applicability in small cohorts, it may still aid prognostication and family counseling in patients with severe PPH.

#### 4.3.2. Neonatal Outcomes

Neonatal birth asphyxia occurred in 18.2% of neonates born to mothers admitted to the ICU versus 4.5% of those whose mothers were not admitted. This difference was clinically important. It likely reflects the earlier delivery timing and greater maternal severity among our ICU patients. Together, these factors increase the rate of preterm birth and contribute to poorer neonatal outcomes.

### 4.4. Strengths of This Study

To our knowledge, this is the first study from Thailand to evaluate ICU utilization, ICU‐related outcomes, associated factors, and a prediction model for ICU admission among patients with PPH following cesarean delivery. A major strength is the use of a large retrospective cohort, which enabled detailed comparison between patients who did and did not require ICU admission. Although the ICU cohort was relatively small, our findings provide insight into ICU utilization and the clinical characteristics associated with critical care requirements among patients with PPH following cesarean delivery.

This study also contributes to the growing body of evidence on maternal critical care from upper‐middle‐income countries. Few studies have specifically examined ICU utilization and ICU‐related outcomes in women with severe PPH in Southeast Asia. By focusing on critical care requirements rather than hemorrhage outcomes alone, this study provides additional insight into the burden and utilization of intensive care resources in severe obstetric hemorrhage.

From a clinical perspective, our findings highlight several characteristics associated with ICU admission, including earlier gestational age, emergency cesarean delivery, general anesthesia, and severe hemorrhage. Recognition of these characteristics may support heightened vigilance, timely escalation of monitoring, multidisciplinary planning, preparedness for massive transfusion, and appropriate critical care support when severe PPH occurs.

### 4.5. Limitations

First, the retrospective design limits causal inference and may be subject to incomplete documentation of important clinical details. In addition, ICU admission decisions were based on clinical judgment, anticipated monitoring needs, and ICU bed availability rather than predefined institutional criteria. Consequently, variation in admission practices may have introduced selection bias and should be considered when evaluating factors associated with ICU admission. Information on planned versus unplanned ICU admission was not consistently documented and therefore could not be reliably analyzed.

Second, our hospital’s limited ICU capacity and absence of a high‐dependency unit have two consequences. Some hemodynamically stable patients requiring close monitoring may have been managed outside the ICU, whereas selected high‐risk patients may have been admitted for short‐term intensive monitoring. This practice may have influenced ICU admission patterns and may limit the generalizability of our findings to institutions with different critical care resources.

Third, the proposed prediction score was derived from a single‐center cohort and has not undergone internal or external validation. Therefore, it should be considered exploratory and requires validation in independent populations before clinical implementation.

Lastly, as a single‐center study, our results may not capture the full spectrum of obstetric care or ICU admission practices in Thailand or other middle‐income settings. Multicenter studies would help validate these findings and improve external generalizability.

## 5. Conclusions

PPH following cesarean delivery remains an important cause of maternal morbidity requiring intensive monitoring and critical care. In this cohort, ICU admission occurred in approximately 7% of patients with PPH and was associated with more severe maternal illness and adverse perioperative outcomes. Factors independently associated with ICU admission included estimated blood loss > 2200 mL, general anesthesia, earlier gestational age, and emergency cesarean delivery. Because some of these factors may reflect the severity of ongoing hemorrhage rather than causal predictors, the findings should be interpreted as markers associated with ICU utilization. These findings improve understanding of critical care utilization among women with severe PPH and may facilitate timely escalation of monitoring, multidisciplinary management, and appropriate critical care when severe hemorrhage occurs.

## Funding

The authors have nothing to report.

## Ethics Statement

This study was performed following approval by the Siriraj Institutional Review Board (approval number Si 161/2021; protocol number 005/2564; February 25, 2021).

## Conflicts of Interest

The authors declare no conflicts of interest.

## Data Availability

The data that support the findings of this study are available upon request from the corresponding author. The data are not publicly available due to privacy or ethical restrictions.
